# Adenosine metabolized from extracellular ATP ameliorates organ injury by triggering A_2B_R signaling

**DOI:** 10.1186/s12931-023-02486-3

**Published:** 2023-07-13

**Authors:** Taha Kelestemur, Zoltán H. Németh, Pal Pacher, Jennet Beesley, Simon C. Robson, Holger K. Eltzschig, György Haskó

**Affiliations:** 1 Department of Anesthesiology, Columbia University, 630 W 168th Street, New York City, NY 10032 USA; 2 Department of Physiology, Faculty of Medicine, Istanbul Medipol University, Istanbul, Turkey; 3 Department of Surgery, Morristown Medical Center, Morristown, NJ 07960 USA; 4 Laboratory of Cardiovascular Physiology and Tissue Injury, National Institute on Alcohol Abuse and Alcoholism, National Institutes of Health, Bethesda, MD USA; 5 Daresbury Proteins Ltd, Sci-Tech Daresbury, Warrington, UK; 6 Department of Anesthesia, Beth Israel Deaconess Medical Center, Boston, MA USA; 7 Department of Anesthesiology, The University of Texas Health Science Center at Houston, Houston, TX USA

**Keywords:** CD39, CD73, A_2B_R, Adenosine, Acute lung injury, Trauma hemorrhagic shock

## Abstract

**Background:**

Trauma and a subsequent hemorrhagic shock (T/HS) result in insufficient oxygen delivery to tissues and multiple organ failure. Extracellular adenosine, which is a product of the extracellular degradation of adenosine 5’ triphosphate (ATP) by the membrane-embedded enzymes CD39 and CD73, is organ protective, as it participates in signaling pathways, which promote cell survival and suppress inflammation through adenosine receptors including the A_2B_R. The aim of this study was to evaluate the role of CD39 and CD73 delivering adenosine to A_2B_Rs in regulating the host’s response to T/HS.

**Methods:**

T/HS shock was induced by blood withdrawal from the femoral artery in wild-type, global knockout (CD39, CD73, A_2B_R) and conditional knockout (intestinal epithelial cell-specific deficient Villin^Cre^-A_2B_R^fl/fl^) mice. At 3 three hours after resuscitation, blood and tissue samples were collected to analyze organ injury.

**Results:**

T/HS upregulated the expression of CD39, CD73, and the A_2B_R in organs. ATP and adenosine levels increased after T/HS in bronchoalveolar lavage fluid. CD39, CD73, and A_2B_R mimics/agonists alleviated lung and liver injury. Antagonists or the CD39, CD73, and A_2B_R knockout (KO) exacerbated lung injury, inflammatory cytokines, and chemokines as well as macrophage and neutrophil infiltration and accumulation in the lung. Agonists reduced the levels of the liver enzymes aspartate transferase and alanine transaminase in the blood, whereas antagonist administration or CD39, CD73, and A_2B_R KO enhanced enzyme levels. In addition, intestinal epithelial cell-specific deficient Villin^Cre^-A_2B_R^fl/fl^ mice showed increased intestinal injury compared to their wild-type Villin^Cre^ controls.

**Conclusion:**

In conclusion, the CD39-CD73-A_2B_R axis protects against T/HS-induced multiple organ failure.

**Supplementary Information:**

The online version contains supplementary material available at 10.1186/s12931-023-02486-3.

## Introduction

Trauma resulting in hemorrhagic shock **(**T/HS) is a medical emergency that occurs when the body loses a considerable amount of blood. It leads to insufficient perfusion of essential organs and tissues due to the reduced blood volume [1–3]. Subsequent coagulopathy, endotheliopathy, microcirculatory dysfunction, inflammatory cell stimulation, and immunological activation result in multiple organ failure (MOF), where the injured organs include the lung, liver, and gut [4–6]. At the cellular level oxygen deficiency leads to the accumulation of lactic acid, inorganic phosphates, and free oxygen radicals [1].

ATP is a nucleotide found in both the extracellular and intracellular spaces where it is involved in important cellular biological processes. Cellular homeostasis is disrupted when intracellular ATP supplies decrease, which can also lead to cell death. ATP or ADP can be released from cells through membrane integrity loss, connexin/pannexin channels, and hormone-transporting vesicles [4, 7–9]. Adenosine is a nucleoside, which can be produced in the extracellular space through the degradation of ATP by ectonucleoside triphosphate diphosphohydrolase-1 (CD39), and ecto-5′-nucleotidase (CD73), which are membrane-associated enzymes. CD39 degrades extracellular ATP or ADP to AMP and CD73 degrades to AMP to adenosine. Also, adenosine can be directly released to the extracellular space via equibilirative nucleoside transporters (ENTs) [10–15]. Extracellular adenosine plays a role in regulating sleep, stroke, vasodilation, and inflammation [16]. Adenosine exerts its effects through binding to P1 or adenosine receptors (A_1_R, A_2A_R, A_2B_R, and A_3_R) and triggering intracellular signaling mechanisms such as up-or down-regulation of cyclic adenosine monophosphate (cAMP) [10, 16–18]. Adenosine receptors are found on many immune cell types such as neutrophils, monocytes, macrophages, dendritic cells, T cells, B cells, and NK cells, as well as on parenchymal cells, where they are involved in the regulation of inflammation [19, 20]. The A_2B_R is expressed on many immune and non-immune cell types, and in general, it has anti-inflammatory and tissue restorative functions [6, 20–33]. We have previously shown that the A_2A_R has a protective role against organ injury after T/HS [5, 6, 34]. However, the role of the A_2B_R and upstream CD39 and CD73 have not been explored. Here we investigated the role of the CD39-CD73-A_2B_R axis in regulating organ injury after T/HS using highly selective antagonists and agonists as well as global and conditional CD39, CD73, and A_2B_R deficient mice.

## Materials and methods

### Ethical statements and animals

All procedures on mice were performed under Columbia University Institutional Animal Care and Use Committee (IACUC) approval number (AABL4551/2021). Adult, male (8-12-week-old n = 4/group) CD39^−/−^, A_2B_R^−/−^, Villin^Cre^-A_2B_AR^fl/fl^, and their WT control Villin^Cre^-A_2B_AR^+/+^ mice were bred at and wild-type C57BL/6J mice obtained from Charles River (Wilmington, MA, USA). CD73^−/−^ (B6.129S1-Nt5e^tm1Lft^/J) mice were purchased from Jackson Laboratory (Bar Harbor, ME, USA). CD73^−/−^ mice were bred and maintained in a specific pathogen-free Columbia University animal facility until being used in experiments. Mice had access to food and water *ad libitum*. They were kept in a room with a 12-h light-dark cycle under nonspecific pathogen-free conditions.

### Drugs

The selective CD39 inhibitor sodium polyoxotungstate (POM1), CD73 inhibitor PSB 12,379 (N6-Benzyl-α,β-methyleneadenosine 5’-diphosphate disodium salt), and adenosine receptor agonist 1-(6-Amino-9 H-purin-9-yl)-1-deoxy-N-ethyl-β-D-ribofuranuronamide (NECA) were from Tocris (Bristol, UK). The CD39 mimic potato apyrase was from Sigma, and recombinant human (rh)CD73 was from Daresbury Proteins (Warrington, UK).

### Study design and induction of trauma hemorrhagic shock

In one set of studies, wild-type mice were randomly assigned into the following groups: trauma/sham shock (T/SS) receiving vehicle (saline), trauma/hemorrhagic shock (T/HS) receiving vehicle (saline), T/HS receiving POM1 (5 mg/kg), T/HS receiving apyrase (125 U/kg), T/HS receiving PSB 12,379 (50 mg/kg), and T/HS receiving rhCD73 (2 mg/kg). In addition, in another group of studies, CD39^−/−^, CD73^−/−^, A_2B_AR ^−/−^ and wild-type type mice were subjected to T/SS or T/HS. Some CD39^−/−^ and CD73^−/−^ mice were pretreated with NECA (0.0025 mg/kg). In pharmacological experiments, mice were pretreated intraperitoneally (30 min before T/SS or T/HS) with various agents.

The mice were given anesthesia using 1% isoflurane and their rectal temperature was maintained between 36.5 and 37.5 °C using a feedback-controlled homeothermic blanket heating system (Sumno-suite, Kent Scientific). Hemorrhagic shock was induced using a fixed-pressure model [34] as described in the Additional data. Initially, a midline laparotomy of 2 cm was performed on the anesthetized mice, which was later closed with a 4 − 0 silk suture (034902, Covetrus, USA). Following this, catheters were placed in the right and left femoral arteries for monitoring blood pressure and blood withdrawal, respectively. A sterile 1-ml syringe with a 30G needle attached to PE-10 tubing filled with 0.2 ml of 1% heparinized saline was used for blood withdrawal, and each mouse received 1U heparin. Blood pressure was monitored using a continuous blood-pressure monitoring system (Powerlab 8/30, ADInstruments, Colorado Springs, CO, USA). After 5 min of baseline blood pressure recording, a drug or vehicle was administered to the mice, followed by inducing shock for a period of 2.5 h, during which the blood pressure was maintained between 28 and 32 mmHg by withdrawing or reinfusing the shed blood. At the end of the shock period, the mice were resuscitated with Ringer’s Lactate at three times the amount of shed blood for 15 min. Three hours after resuscitation, the mice were euthanized and bronchoalveolar lavage fluid (BALF), blood, and tissue samples were collected. T/SS animals were exposed to the same procedures as other animals except for blood withdrawal (Additional file 1: Fig. S1).

### Tissue preparation for evaluating neutrophil sequestration, mouse cytokine array, and western blot analysis

Lung, liver, kidney, and gut samples were pooled from 4 mice in the same group, homogenized, sonicated, and treated with a 1X RIPA Buffer with Protease/phosphatase inhibitor cocktail (P8340, Sigma, USA), and the resulting homogenate was centrifuged at 13.000 x *g* for 10 min at 4 °C. Total protein content was determined by using Qubit 4.0 Fluorometer (Thermo Fisher, USA) as per the manufacturer’s instructions.

### Collection of BALF samples and determination of BALF ATP, Adenosine, and cAMP levels

Three hours after the end of resuscitation the mice were euthanized, and the trachea was isolated for collecting BALF samples. Briefly, after a small incision a syringe with a 23G needle filled with 1 ml of sterile saline was inserted into the trachea. After centrifugation (1500 x *g* for 15 min at 4 °C) the supernatant was used for the assays [34]. ATP (ab83355, Abcam, USA) and cAMP (KGE012B, R&D Systems, USA) in BALF were measured by a colorimetric assay. Adenosine was measured by a fluorometric assay (ab211094, Abcam, USA) according to the manufacturer’s instructions.

### Lung permeability measurements

Mice were re-anesthetized with isoflurane 3 h after the end of resuscitation. Evans blue dye (EBD) technique was used to determine lung permeability. EBD was administered through the tail vein, and about 1 ml of blood was withdrawn from the tail artery five minutes later. The supernatant of BALF was assayed at 620 nm spectrophotometrically. The amount of Evans blue dye in the BALF was then expressed as a proportion of the amount in the plasma [5, 34].

### Determination of pulmonary neutrophil sequestration

To evaluate neutrophil sequestration in the lung following T/SS or T/HS, myeloperoxidase (MPO) activity was assessed using an MPO activity kit (MAK068, Sigma, USA). The supernatant of lung lysates was assayed according to the manufacturer’s instructions [34].

### Mouse cytokine array

To examine cytokine and chemokine expression in lung samples, a total of 200 µg protein was analyzed using the Proteome Profiler Mouse Cytokine Array Panel A Kit (ARY006, R&D Systems, USA). The expression of cytokines and chemokines was determined densitometrically using Fiji software after subtracting the negative control’s average signal. Values were expressed as % of T/SS [35].

### Immunofluorescence staining

Macrophage staining was performed as previously described [35]. Lung section (5 μm) were fixed in 4% paraformaldehyde in PBS, washed, and immersed for 30 min in 0.1 M PBS containing 0.3% Triton-X-100 (PBS-T)/10% normal goat serum. Sequentially 2 lung sections from each sample were incubated overnight at 4 °C with AlexaFlour 488-conjugated monoclonal mouse anti-F4/80 (53-4801-82, Thermo Fisher, USA). The following day, sections were incubated with 4′,6-diamidino-2-phenylindole (DAPI) (D9542, Sigma, USA) at room temperature for 5 min. Sections were analyzed using confocal laser scanning microscopy (Zeiss LSM 900, Jena, Germany). Eight different regions of interest (ROI) from the sections were calculated. The percentage of macrophages in the lung tissue was calculated by dividing the results obtained from T/HS by the results of T/SS and then multiplying the quotient by 100.

### Western blot

CD39, CD73, A_2B_R, phosphorylated-Phosphatase and Tensin (p-PTEN) and matrix metalloproteinase-9 (MMP-9) protein levels after T/SS or T/HS were determined using western blotting of pooled samples run three times. Thirty micrograms of protein per sample were size-fractioned using 4–20% Mini-Protean TGX Stain-Free (4,568,093, Bio-Rad, Life Sciences Research) electrophoresis gel and then transferred to a PVDF membrane (1,620,174, Bio-Rad, Life Sciences Research) using Mini Trans-Blot Electrophoretic Transfer System (1,703,930, Bio-Rad, Life Sciences Research). The membranes were first blocked with a blocking solution composed of 5% non-fat dry milk in 50 mM Tris-buffered saline containing 0.1% Tween 20 (TBS-T) for 1 h at room temperature. Following this, the membranes were washed with 50 mM TBS-T and then incubated overnight with a rabbit monoclonal anti-CD39 (ab223842, Abcam, USA), rabbit polyclonal anti-CD73 (ab175396, Abcam, USA), rabbit polyclonal anti-A_2B_R (ab229671, Abcam, USA), rabbit polyclonal p-PTEN (9551, Cell Signaling, USA) and rabbit polyclonal anti-MMP-9 (ab38898) at a dilution 1:1000, 1:1000, 1:500, 1:1000 and 1:2000 respectively. The following day, the membranes were washed with TBS-T and then incubated with horseradish peroxidase (HRP)-conjugated goat anti-rabbit secondary antibody (ab97051, Abcam, USA) at a dilution of 1:5000 in the blocking solution for 2 h at room temperature. To ensure the protein loading was consistent, the membranes were stripped and re-probed with an HRP-conjugated anti-ß actin antibody (ab20272, Abcam, USA). The membranes were then developed using Clarity Western ECL Substrate Kit (1,708,280, Bio-Rad, Life Sciences Research) [34].

### Measurement of AST and ALT levels

Plasma samples were analyzed to determine the levels of aspartate aminotransferase (AST) and alanine aminotransferase (ALT). To do this, the samples were diluted with AST (TR70121 Thermo Fisher, USA) and ALT (TR71122 Thermo Fisher, USA) reagents at a 1:10 ratio, and the resulting light signal was measured using a spectrophotometer at 340 and 405 nm [34].

### Histopathological assessment of lung and intestinal injury

Sections of lung and gut were cut at 5 μm thickness, stained with the hematoxylin-eosin (H&E) method, and scanned with a Lecia AT2 slide scanner (Leica, USA). Lung sections were evaluated histopathologically in terms of the parameters: 1: neutrophils in the alveolar spaces, 2: neutrophils in the interstitial space, 3: hyaline membranes, 4: proteinaceous debris filling in the airspaces, 5: alveolar septal thickening by a blind observer [36]. Ileum samples were evaluated histopathologically in terms of the parameters: 1: desquamation and necrosis of upper 1/3 villi, 2: progressive peel off mid of villi, 3: peel of lower 1/3 villi and necrosis of cript cells, 4: necrosis of 2/3 cript cells, 5: complete loss of basal cripts.

### Determination of IL-10 and IL-6 levels by enzyme-linked immunosorbent assay

IL-6 and IL-10 levels were measured in plasma samples using enzyme-linked immunosorbent assay (ELISA) kits (DY406, DY417; R&D Systems Minneapolis, USA) according to the manufacturer’s instructions [34].

### Statistical analysis

The Shapiro-Wilk test was used to determine the normality of the data in each group, which demonstrated a normal distribution. Statistical analysis was conducted using Graph-Pad Prism (San Diego, CA, USA). One-way ANOVA or t-test was performed followed by Tukey’s test as appropriate. The results were presented as mean ± S.D. values, and a p-value of less than 0.05 was considered statistically significant.

## Results

### T/HS increases CD39, CD73, and A_2B_R expression in lung, liver, kidney, and gut

**Fig. 1:**
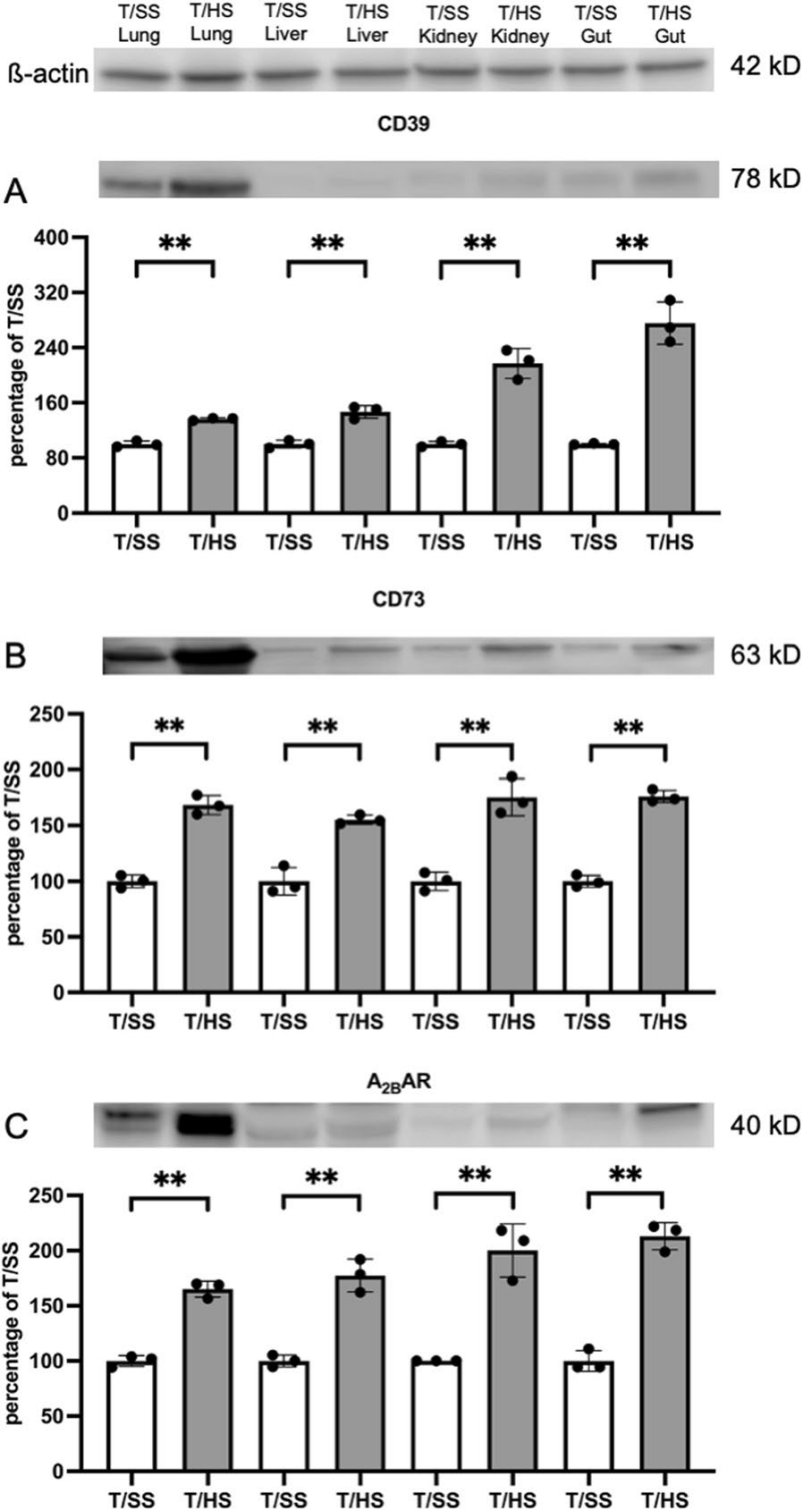
T/HS increases CD39, CD73, and A_2B_AR expression in lung, liver, kidney, and gut. **A–C** CD39, CD73, and A_2B_AR expression were evaluated using western blotting of protein extracts. Data are mean ± S.D. (n = 4/group). **p < 0.01 compared with T/SS

T/HS increased the expression of CD39, CD73, and the A_2B_R in the lung, liver, kidney, and gut when compared to T/SS (Fig. 1, A-C). Of all the organs investigated, CD39, CD73 and the A_2B_R expression was highest in the lung. Thus in subsequent experiments, our main but not exclusive focus was on lung injury.

### Role of CD39, CD73, and A_2B_R in regulating ATP, adenosine, and cAMP levels after T/HS

**Fig. 2:**
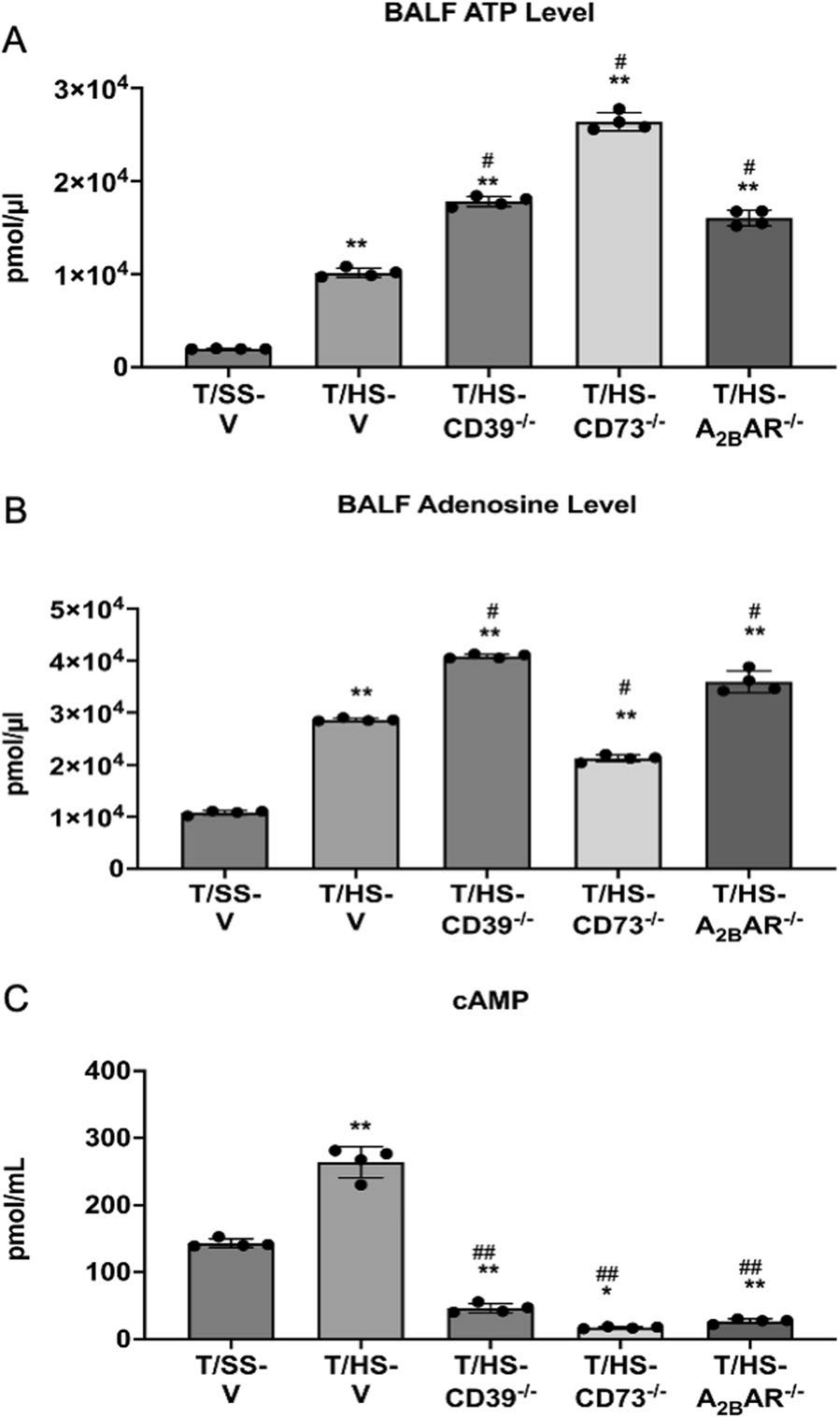
ATP (**A**), adenosine (**B**), and cAMP (**C**) levels in BALF following T/HS in the CD39, CD73, and A_2B_AR KO samples. ATP and cAMP were measured colorimetrically and adenosine was measured by a fluorometric assay. Data are mean ± S.D. (n = 4/group) *p < 0.05 compared with T/SS-V, **p < 0.01 compared with T/SS-V, ^#^p < 0.05 compared with T/HS-V, ^##^p < 0.01 compared with T/HS-V

We found that T/HS increased ATP, adenosine, and cAMP levels in BALF samples (Fig. 2A–C). CD39, CD73, and A_2B_R KO further increased T/HS-induced ATP and adenosine (Fig. 2A, B). T/HS increased cAMP compared to T/SS and cAMP levels decreased in CD39, CD73, and A_2B_R KO mice in T/HS BALF (Fig. 2C).

### CD39, CD73, and the A_2B_R suppress lung permeability and neutrophil infiltration

**Fig. 3:**
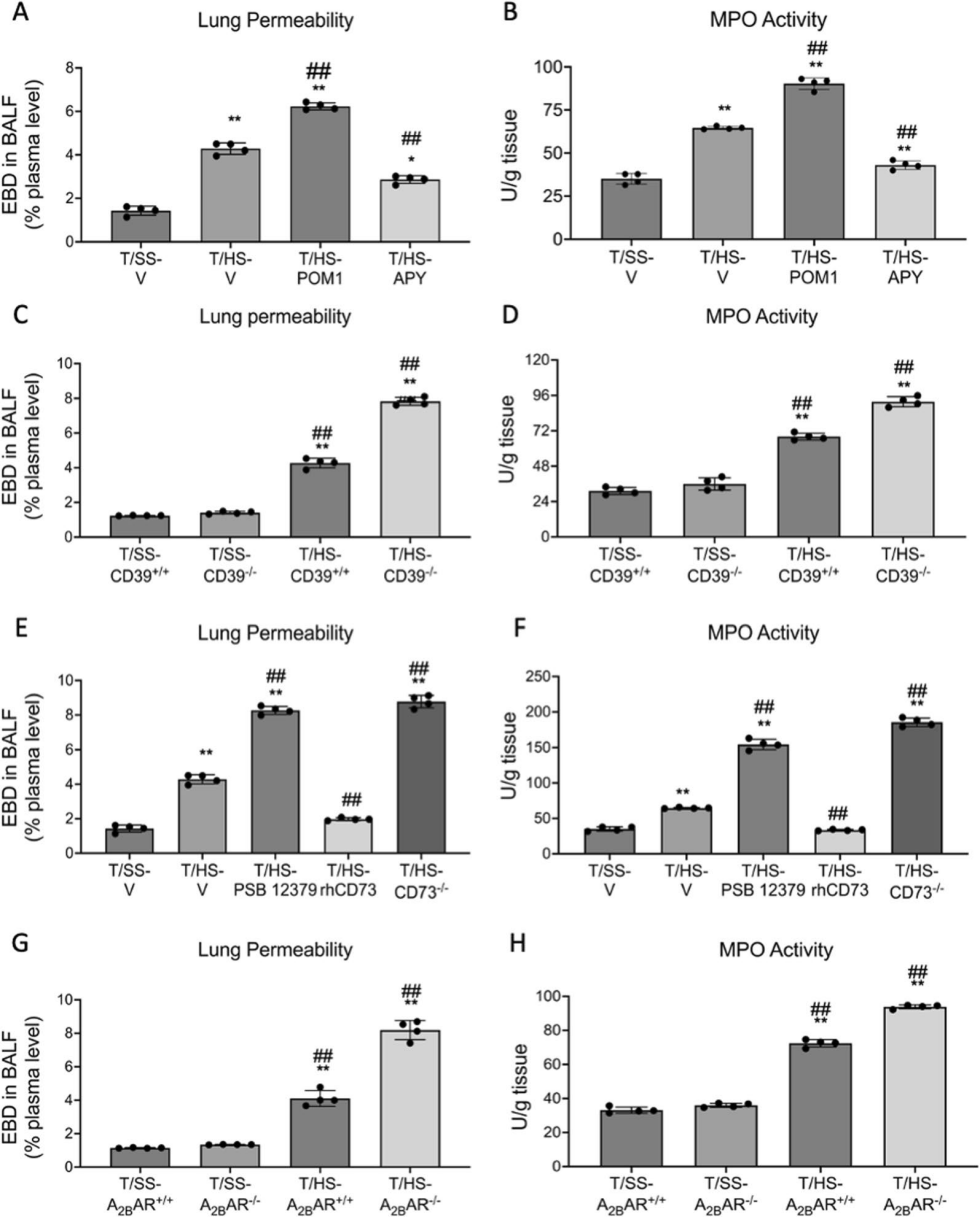
CD39, CD73, and A_2B_R regulation of lung permeability and MPO activity. Lung permeability was determined using the EBD method (**A**, **C**, **E**, **G**) and MPO activity as a surrogate for neutrophil sequestration (**B**, **D**, **F**, **H**) was determined spectrophotometrically. Data are mean ± S.D. (n = 4/group). *p < 0.05 compared with corresponding T/SS, **p < 0.01 compared with corresponding T/SS, ^#^p < 0.05 compared with corresponding T/HS-V, ^##^p < 0.01 compared with corresponding T/HS

**Fig. 4:**
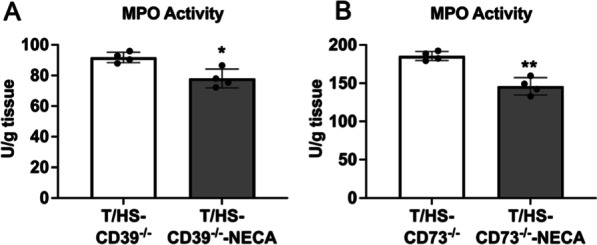
Effect of NECA on lung MPO activity in CD39^−/−^ and CD73^−/−^ mice. MPO activity was determined from the lung spectrophotometrically (**A**, **B**). Data are mean ± S.D. (n = 4/group). *p < 0.05 compared with T/HS, **p < 0.05 compared with T/HS

**Fig. 5:**
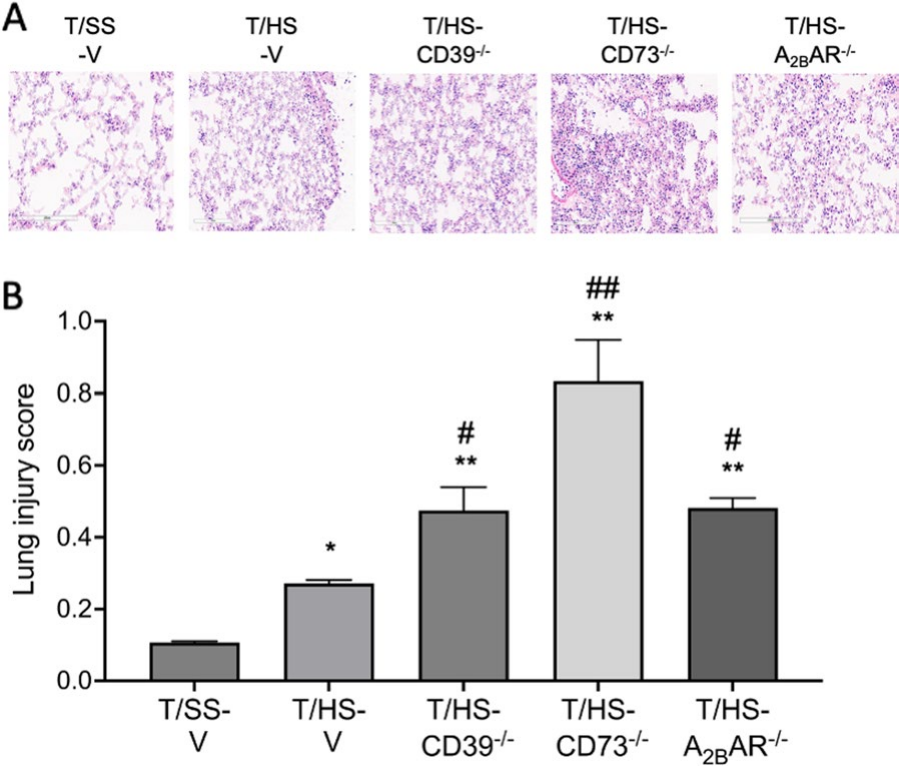
Lung injury was induced by T/HS. Representative images of H&E-stained lung samples (**A**). Pulmonary injury was evaluated using 5 independent parameters (Neutrophils in the alveolar space, neutrophils in the interstitial space, hyaline membranes, proteinaceous debris filling the airspaces and alveolar septal thickness) (**B**). Data are mean ± S.D. (n = 4/group). *p < 0.05 compared with T/HS, **p < 0.05 compared with T/HS, ^#^p < 0.05 compared with T/HS-V, ^##^p < 0.01 compared with T/HS-V

Treatment with the CD39 antagonist POM1 exacerbated and treatment with the CD39-like enzyme apyrase moderated T/HS-induced lung injury and neutrophil sequestration after T/HS (Fig. 3A, B). KO of CD39, CD73, and the A_2B_R increased lung permeability and neutrophil sequestration (Fig. 3C–H). The CD73 antagonist PSB 12,379 increased lung injury and neutrophil infiltration while rhCD73 decreased these parameters (Fig. 3E, F). We then examined whether adenosine receptor stimulation was able to rescue the increased injury in CD39^−/−^ and CD73^−/−^ mice. NECA pretreatment decreased neutrophil infiltration in CD39^−/−^ and CD73^−/−^ mice after T/HS (Fig. 4A, B). The lung injury score increased in T/HS vs. T/SS mice and was further increased in the CD39, CD73, and A_2B_R KO mice (Fig. 5A, B).

### Regulation of lung cytokine levels by CD39, CD73, and the A_2B_R

**Fig. 6:**
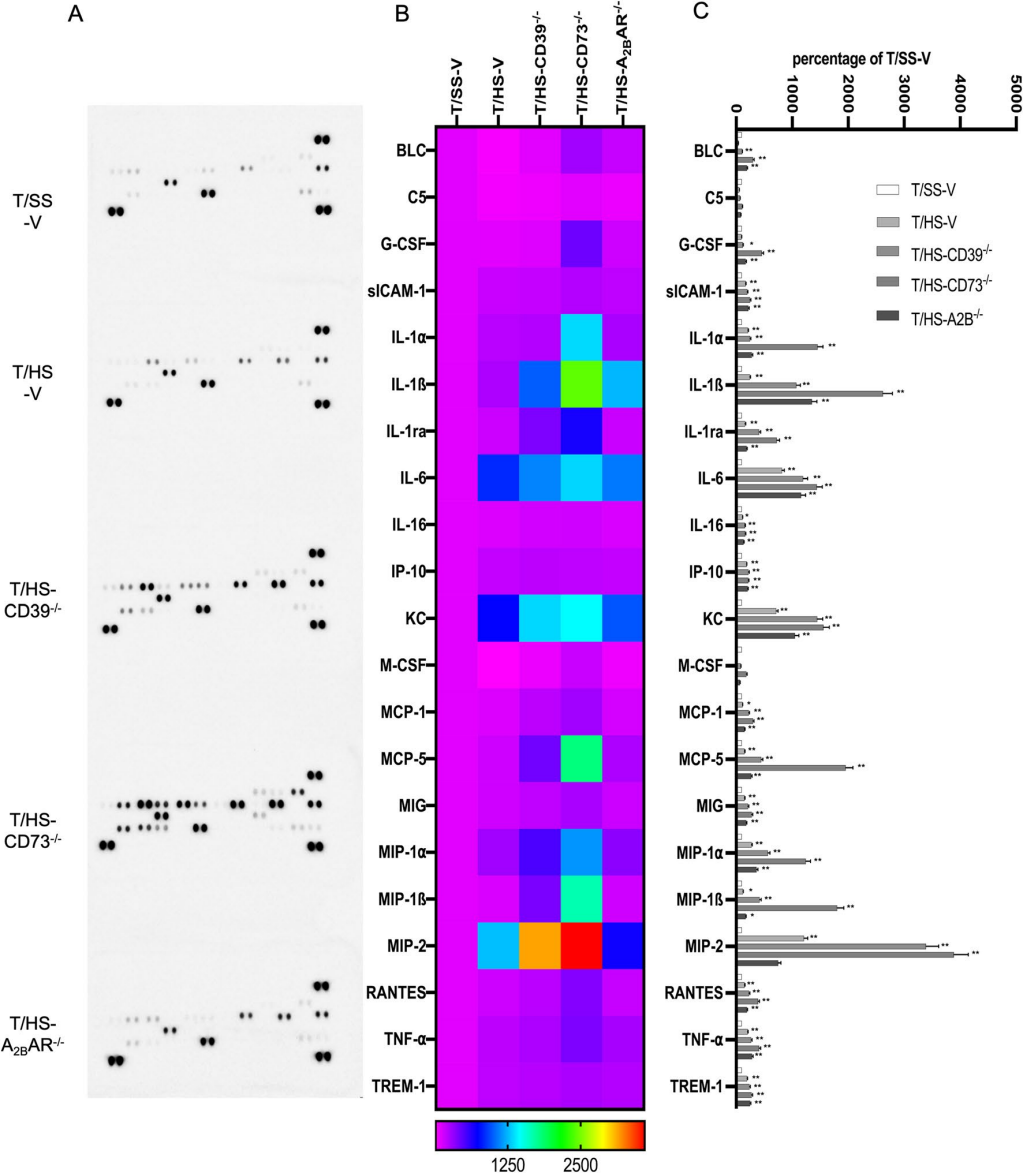
Quantification of pro- and anti-inflammatory cytokines and chemokines in the lung. Representative images of cytokine-chemokine arrays that were used to interrogate cytokine expression (**A**). Proteome Profiler Mouse Cytokine-Chemokine array was used to analyze cytokines in mice subjected to T/HS. Data were visualized by transforming them into a heat map **B** and also expressed as a bar graph (**C**). Data are mean ± S.D. (n = 4/group). *p < 0.05 compared with T/HS, **p < 0.05 compared with T/HS, ^#^p < 0.05 compared with T/HS-V, ^##^p < 0.01 compared with T/HS-V

Using an antibody array, we assessed the relative expression levels of 40 different cytokines and chemokines including CXCL13/BLC/BCA-1, C5a, G-CSF, GM-CSF, CCL1/I-309, CCL11/Eotaxin, ICAM-1, IFN-gamma, IL-1 alpha/IL-1F1, IL-1 beta/IL-1F2, IL-1ra/IL-1F3, IL-2, IL-3, IL-4, IL-5, IL-6, IL-7, IL-10, IL-12 p70, IL-13, IL-16, IL-17, IL-23, IL-27, CXCL10/IP-10, CXCL11/I-TAC, CXCL1/KC, M-CSF, CCL2/JE/MCP-1, CCL12/MCP-5, CXCL9/MIG, CCL3/MIP-1 alpha, CCL4/MIP-1 beta, CXCL2/MIP-2, CCL5/RANTES, CXCL12/SDF-1, CCL17/TARC, TIMP-1, TNF-alpha, and TREM-1 in the lung (Fig. 6A). We found that levels of pro-inflammatory cytokines and chemokines (BLC, C5, G-CSF, SICAM-1, IL-1α, IL-1ß, IL-1ra, IL-6, IL-16, IP-10, KC, M-CSF, MCP-1, MCP-5, MIG, MIP-1α, MIP-1ß, MIP-2, RANTES, TNF- α and TREM-1) increased in the CD39, CD73, and A_2B_Rs KO mice in the lung after T/HS (Fig. 6B, C).

### CD39, CD73, and A_2B_R moderate macrophage accumulation in the lung

**Fig. 7:**
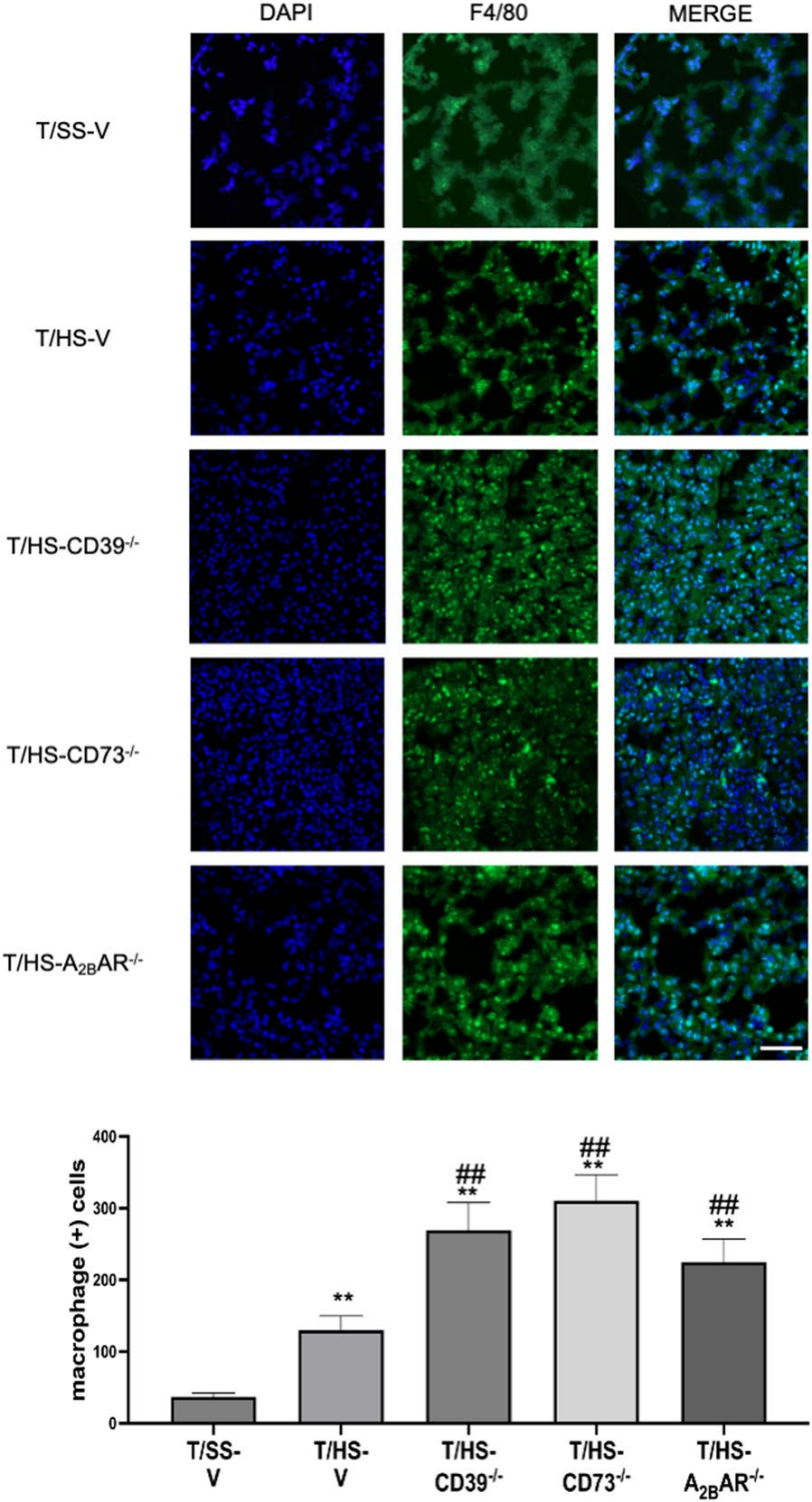
Macrophage accumulation in the lung after T/HS. Accumulated macrophages were determined by F4/80 immunostaining in the lung and representative images are shown as well as averages and means of macrophage counts in the various groups. Data are mean ± S.D. (n = 4/group). *p < 0.05 compared with T/HS, **p < 0.05 compared with T/HS, ^#^p < 0.05 compared with corresponding T/HS-V, ^##^p < 0.01 compared with corresponding T/HS

CD39, CD73, and A_2B_R deficiency all increased macrophage accumulation following T/HS, as indicated by immunofluorescence (Fig. 7).

### Effect of CD39, CD73 and A_2B_R KO on the expression of survival-related proteins

**Fig. 8:**
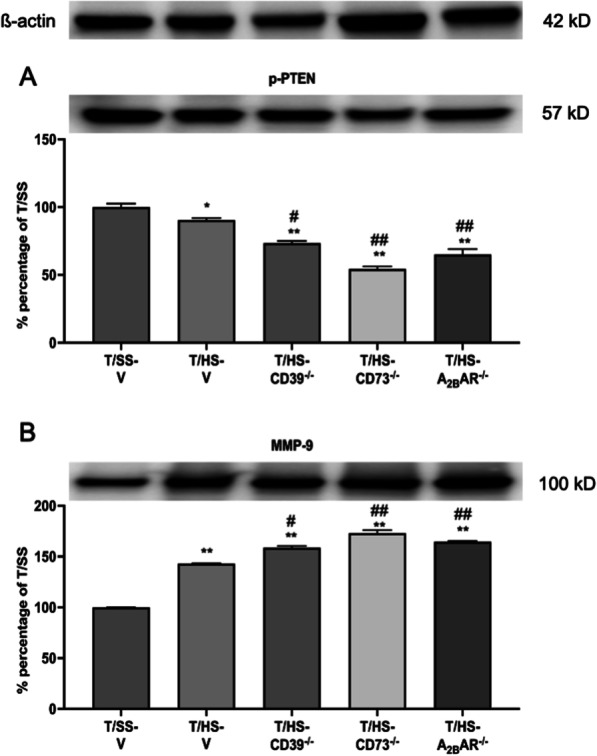
Expression levels of survival-related kinases after T/HS. p-PTEN **A** and MMP-9 **B** expression levels were determined using western blot. Data are mean ± S.D. (n = 4/group). *p < 0.05 compared with T/HS, **p < 0.05 compared with T/HS, ^#^p < 0.05 compared with corresponding T/HS-V, ^##^p < 0.01 compared with corresponding T/HS

To investigate the role of CD39, CD73, and the A_2B_R in regulating the expression of survival-related proteins, we analyzed p-PTEN and MMP-9 expression after T/HS. CD39, CD73, and A_2B_Rs KO all decreased p-PTEN (Fig. 8A) and increased MMP-9 (Fig. 8B) expression levels in lung tissue.

### Regulation of intestinal injury by CD39, CD73, and the A_2B_R

T/HS increased gut injury compared to T/SS (Additional file 2: Fig. S2). Global CD39, CD73, and A_2B_R deficiency failed to influence the extent of gut injury in T/HS mice (Fig. 2). However, Villin^Cre^-A_2B_AR^fl/fl^ mice had increased gut injury when compared to their WT control Villin^Cre^-A_2B_AR^+/+^ mice after T/HS. (Additional file 3: Fig. S3).

### Role of CD39, CD73 and A_2B_R in regulating T/HS-induced liver injury

Apyrase or rhCD73 treatment suppressed T/HS-enhanced AST and ALT levels, indicating decreased liver injury, while POM1 and PSB 12,379 increased AST and ALT (Additional file 4: Fig. S4A, B). In addition, KO of CD39, CD73, and A_2B_Rs led to elevated AST and ALT levels (Additional file 4: Fig. S4C, D).

### Role of CD39, CD73 and A_2B_Rs in regulating plasma IL-6 and IL-10 levels

As compared to vehicle-treated T/HS mice, POM1-treated mice had higher plasma IL-6 levels and similar plasma IL-10 levels. Also, apyrase-treated T/HS mice had lower plasma IL-6 levels and increased plasma IL-10 levels compared to vehicle-treated T/HS mice (Additional file 5: Fig. S5A, B). CD39^−/−^ mice after T/HS had increased IL-6 and decreased IL-10 compared to wild-type controls (Additional file 5: Fig. S5C, D).

When compared to vehicle-treated T/HS mice, PSB 12,379-treated T/HS animals had increased plasma IL-6 and reduced IL-10 levels and rhCD73-treated T/HS mice had decreased plasma IL-6 and increased plasma IL-10 (Figures E6E and E6F). Plasma IL-6 was increased and IL-10 decreased in CD73^−/−^ compared to wild-type T/HS mice (Additional file 5: Fig. S5E, F). A_2B_AR^−/−^ mice exhibited higher plasma IL-6 levels while having lower plasma IL-10 levels compared to the A_2B_R^+/+^ group subjected to T/HS (Additional file 5: Fig. S5G, H).

## Discussion

We recently demonstrated that exogenous stimulation of the A_2A_R decreases organ injury after T/HS. In addition, we found that inhibition or KO of the A_2A_R leads to increased lung permeability, MPO level, and augmented liver enzymes [34]. Our current studies demonstrate for the first time that ATP is released into the extracellular space during T/HS. In agreement with previous studies implicating extracellular ATP as the primary source for adenosine generation in ischemia of particular organs [4, 7–9], our studies targeting CD39 and CD73 demonstrate that ATP is also the likely source of extracellular adenosine following T/HS, a condition whose pathophysiology is much more complex than that of ischemia of the various organs. In addition, we demonstrate for the first time that the A_2B_R activated by endogenous adenosine protects organs from T/HS-induced organ injury.

CD39 is a transmembrane protein found in the spleen, thymus, lung, and placenta and is largely linked with immune cell populations and endothelial cells [10, 37, 38]. Several proinflammatory cytokines, oxidative stress, and hypoxia affect the CD39 expression [39]. Studies indicate that CD39 is organ protective against ischemia/reperfusion injury, sepsis, and heart disease [40–47]. For example, in CD39 deficient mice the organ injury and inflammation that followed cardiac [44], renal [41], hepatic [18, 42], and intestinal [48] ischemia-reperfusion injury were more severe than in the corresponding wild-type mice. A common thread in these studies was that exogenous supplementation with potato apyrase reversed the increased ischemic organ injury of CD39 deficient mice. Potato apyrase is widely used as a CD39 mimic, because it has an amino acid sequence that is highly homologous to CD39, particularly within its four apyrase-conserved regions [49]. Given that T/HS causes global ischemia-reperfusion injury, our results demonstrating protective effects for CD39 in T/HS correspond well with earlier results with ischemia-reperfusion injury of the various organs. Given the commonalities of mechanisms for the injury of the various organs, it will be of interest to determine whether protection in the various models can be traced back to one particular cell type expressing CD39 or whether protection depends on different cell types based on the organ.

CD73 regulates multiple events such as cellular hemostasis and tissue injury and is found in a variety of tissues, including the colon, brain, kidney, liver, lung, and heart [50]; on leukocytes derived from peripheral blood, spleen, lymph nodes, thymus, and bone marrow [50]; as well as on endothelium [51]. The expression and function of this enzyme are upregulated under hypoxic conditions [52, 53], and by several proinflammatory mediators, such as transforming growth factor (TGF)-β, interferons (IFNs), TNF-α, IL-1β, and prostaglandin E_2_ [54, 55]. Similar to CD39, CD73 is also organ protective against hypoxic and ischemic conditions [50, 53, 56]. The increased vascular permeability and polymorphonuclear neutrophil extravasation noted in hypoxic CD73 deficient mice were reversed by stimulating ARs by exogenous administration of 5′-(N-ethylcarboxamido) adenosine (NECA), a general AR agonist, or by exogenous reconstitution with a soluble CD73-like nucleotidase [50]. In this respect, our data with the NECA-mediated “rescue” of the increased neutrophil infiltration of lungs observed in CD73 (and CD39) deficient mice indicate that CD73 together with CD39 deliver adenosine to adenosine receptors to dampen inflammation and injury in T/HS.

The A_2B_R activates intracellular signaling mechanisms such as cAMP [57]. Elevated intracellular levels of cAMP are frequently linked to anti-inflammatory outcomes, including the synthesis of IL-10, the inhibition of leukocyte infiltration, and pro-inflammatory cytokines [58]. We have previously shown that A_2B_R-deficient mice have higher mortality and increased pro-inflammatory cytokines such as IL-6, TNF-α and MIP-2 in sepsis [59]. Also, posttreatment with the A_2B_R agonist BAY 60-6583 decreases lung permeability but not neutrophil infiltration into the lung in a rat model of T/HS [6]. The current studies confirm the generally protective role of A_2B_R in a global ischemia-reperfusion model.

There are several outstanding questions that require further studies to answer. In our view, the most pressing of these are (1) what organs and cells release ATP in T/HS, (2) what are the release mechanisms, and (3) which cell types expressing CD39, CD73, and A_2B_R mediate protection.

In conclusion, our work indicates the organ-protective effects of the purinergic system in a T/HS model. The results point to the purinergic system as a therapeutic target to treat traumatic hemorrhagic shock.

## Supplementary Information

**Additional file 1: Fig. S1. **Experimental design. Mice are exposed to T/SS or T/HS for 2.5 h. Mice received vehicle, agonist, or antagonist 30 min before shock induction. After a 15-min resuscitation period and a subsequent observation period of 3 hours, mice were euthanized and tissues were collected

**Additional file 2: Fig. S2. **Assessment of intestinal injury after T/HS. Effects of CD39, CD73 and A2BR deficiency are shown. Data are mean ± S.D. (n = 4/group). *p < 0.05 compared with T/HS, **p < 0.05 compared with T/HS.

**Additional file 3. Fig. S3.** Determination of intestinal injury after T/HS in IEC-specific A_2B_R deficient mice. Results with IEC-specific Villin^Cre^-A_2B_R^fl/fl^ mice and control are shown. Data are mean ± S.D. (n = 4/group). **p < 0.05 compared with T/HS-Villin^Cre^-A_2B_AR^+/+^.

**Additional file 4. Fig. S4.** CD39, CD73, and A_2B_R regulation of liver enzymes. Aspartate aminotransferase (AST) (**A**, **C**) and alanine aminotransferase (ALT) (**B**, **D**) levels were determined from plasma spectrophotometrically. Data are mean ± S.D. (n = 4/group). *p < 0.05 compared with T/HS, **p < 0.05 compared with T/HS, ^#^p < 0.05 compared with corresponding T/HS-V, ^##^p < 0.01 compared with corresponding T/HS.

**Additional file 5. Fig. S5. **Quantification of cytokine levels in plasma. IL-6 (**A**, **C**, **E**, **G**) and IL-10 (**B**, **D**, **F**, **H**) in blood 3 h following resuscitation were determined using ELISA. Data are mean ± S.D. (n = 4/group). *p < 0.05 compared with T/HS, **p < 0.05 compared with T/HS, ^#^p < 0.05 compared with corresponding T/HS-V, ^##^p < 0.01 compared with corresponding T/HS.

## Data Availability

Not applicable.
