# Supplementary figures and images for: Adenosine metabolized from extracellular ATP ameliorates organ injury by triggering A2BR signaling
Source: Respir Res. 2023 Jul 13;24:186. doi: 10.1186/s12931-023-02486-3 (PMC10339538; doi:10.1186/s12931-023-02486-3)

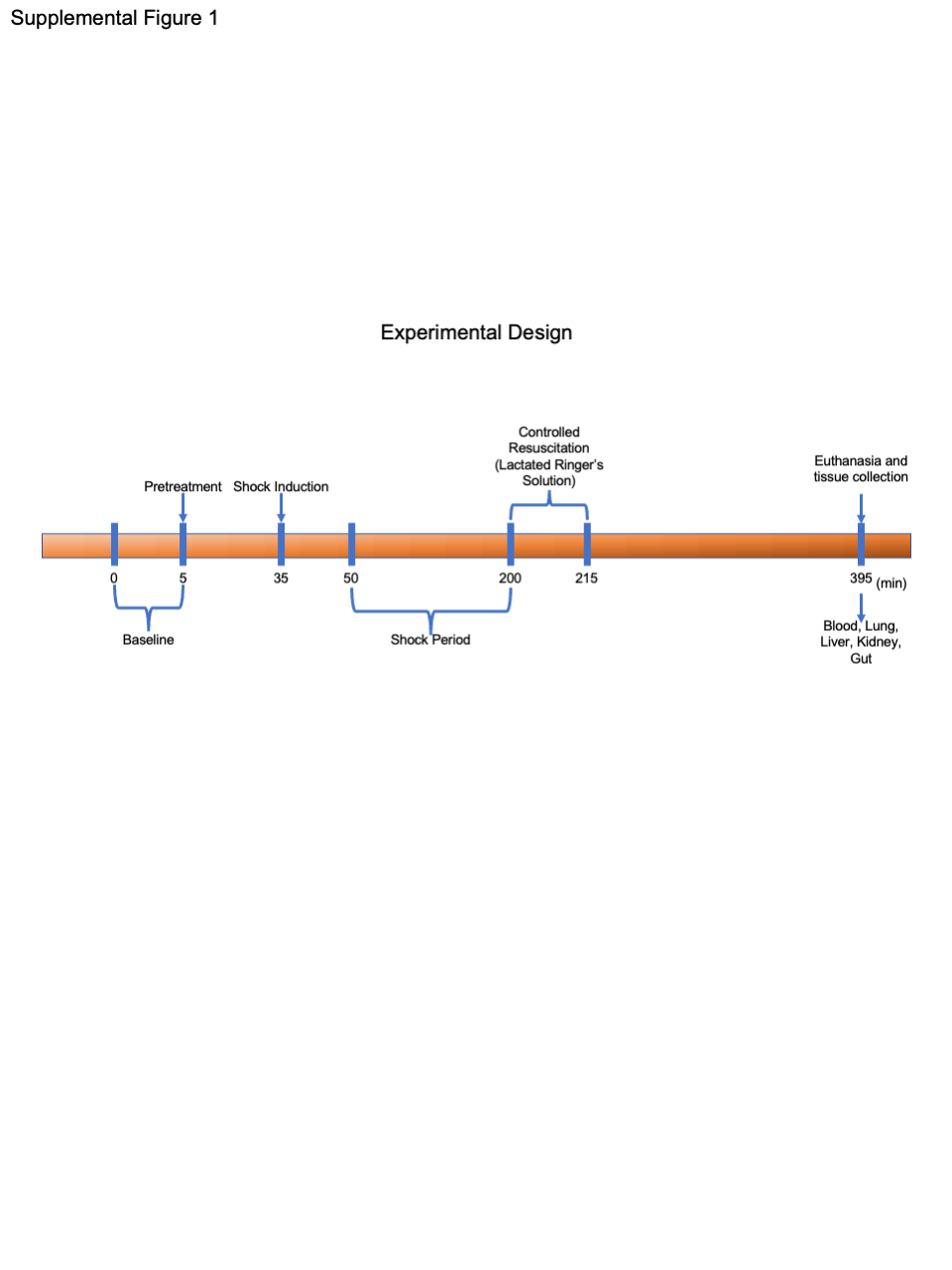

Supplement: Supplementary file 1 — Additional file 1: Fig. S1. Experimental design. Mice are exposed to T/SS or T/HS for 2.5 h. Mice received vehicle, agonist, or antagonist 30 min before shock induction. After a 15-min resuscitation period and a subsequent observation period of 3 hours, mice were euthanized and tissues were collected [file 12931_2023_2486_MOESM1_ESM.tiff]

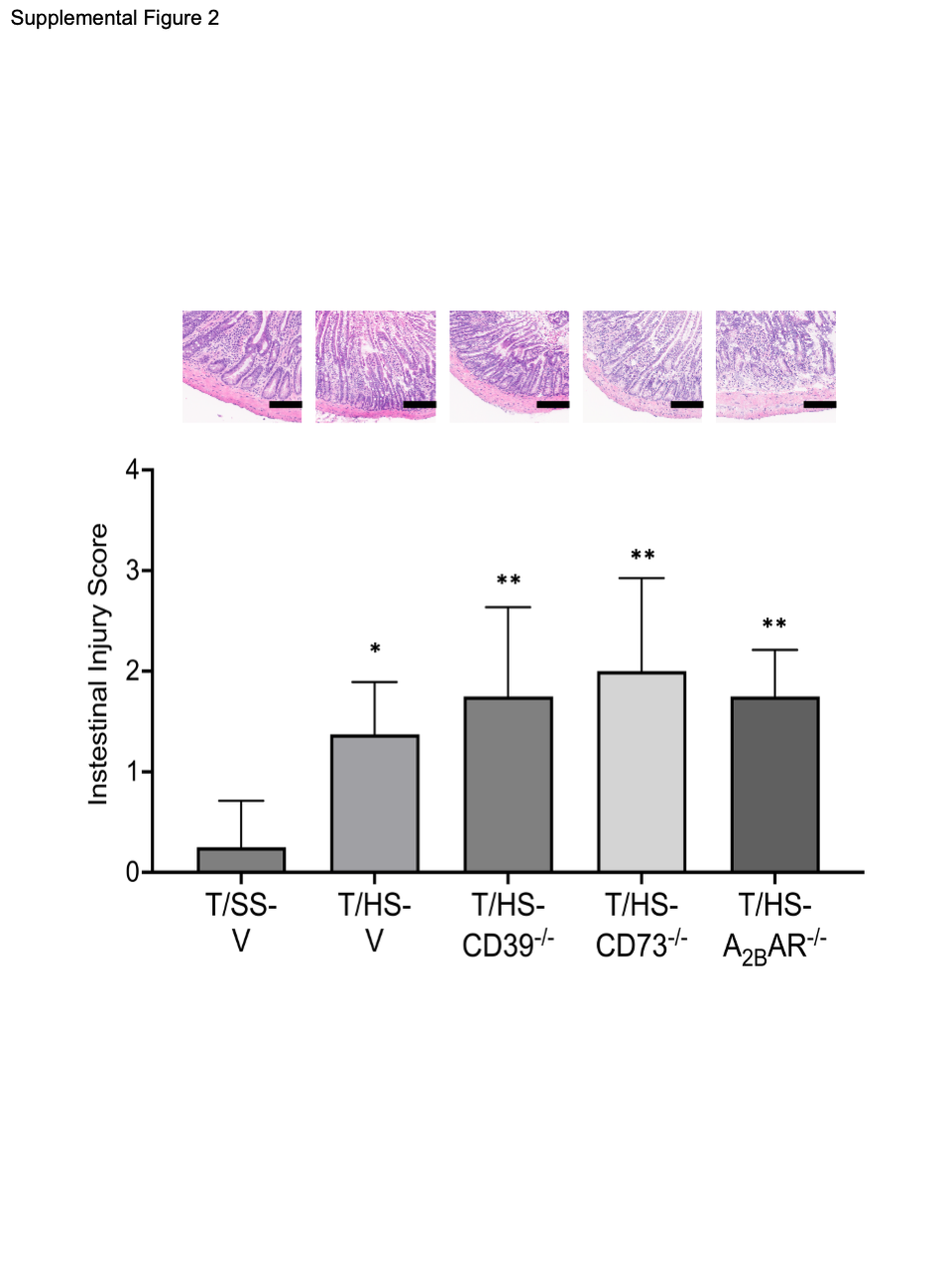

Supplement: Supplementary file 2 — Additional file 2: Fig. S2. Assessment of intestinal injury after T/HS. Effects of CD39, CD73 and A2BR deficiency are shown. Data are mean ± S.D. (n = 4/group). *p < 0.05 compared with T/HS, **p < 0.05 compared with T/HS. [file 12931_2023_2486_MOESM2_ESM.tiff]

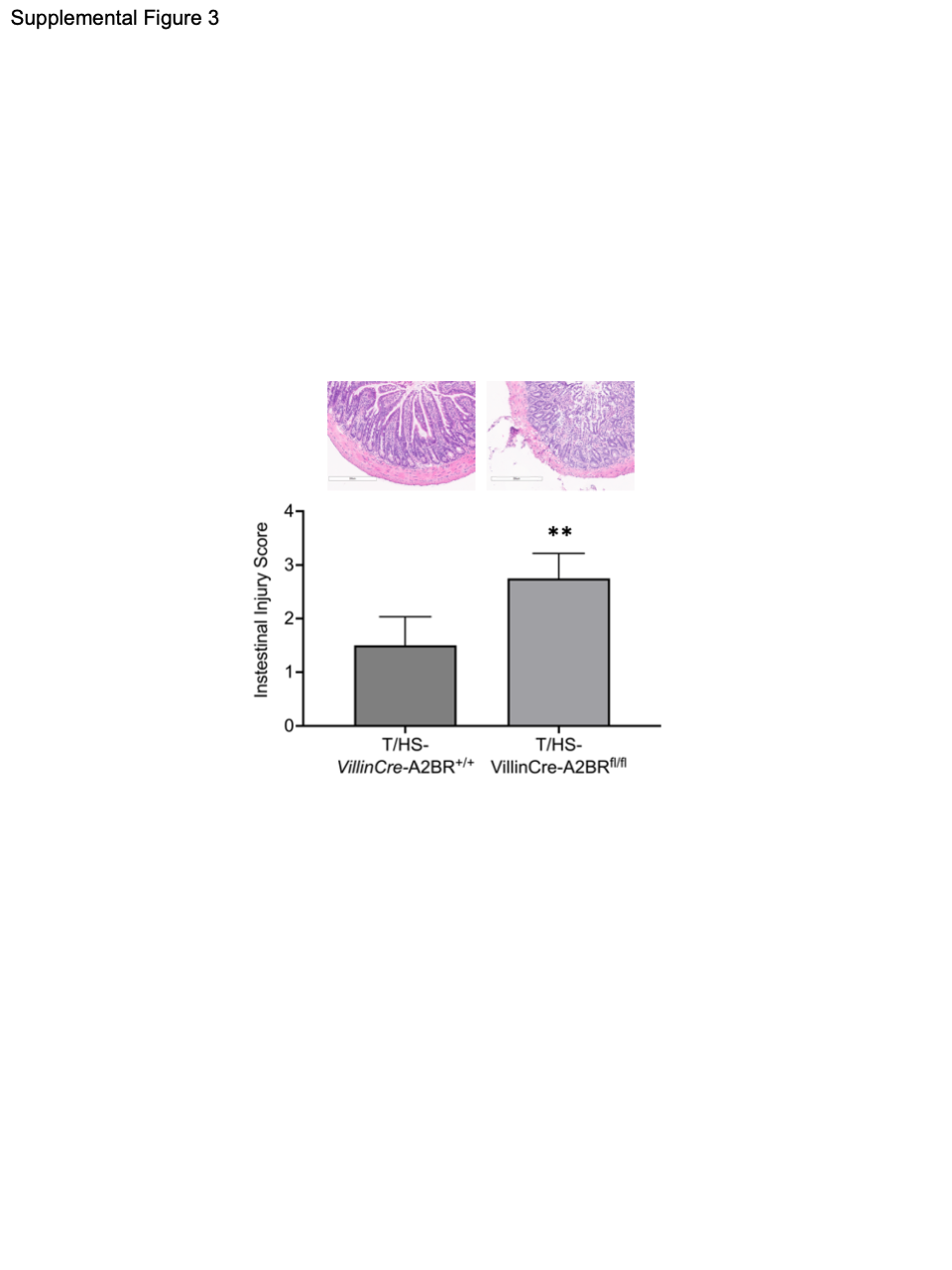

Supplement: Supplementary file 3 — Additional file 3. Fig. S3. Determination of intestinal injury after T/HS in IEC-specific A2BR deficient mice. Results with IEC-specific VillinCre-A2BRfl/fl mice and control are shown. Data are mean ± S.D. (n = 4/group). **p < 0.05 compared with T/HS-VillinCre-A2BAR+/+. [file 12931_2023_2486_MOESM3_ESM.tiff]

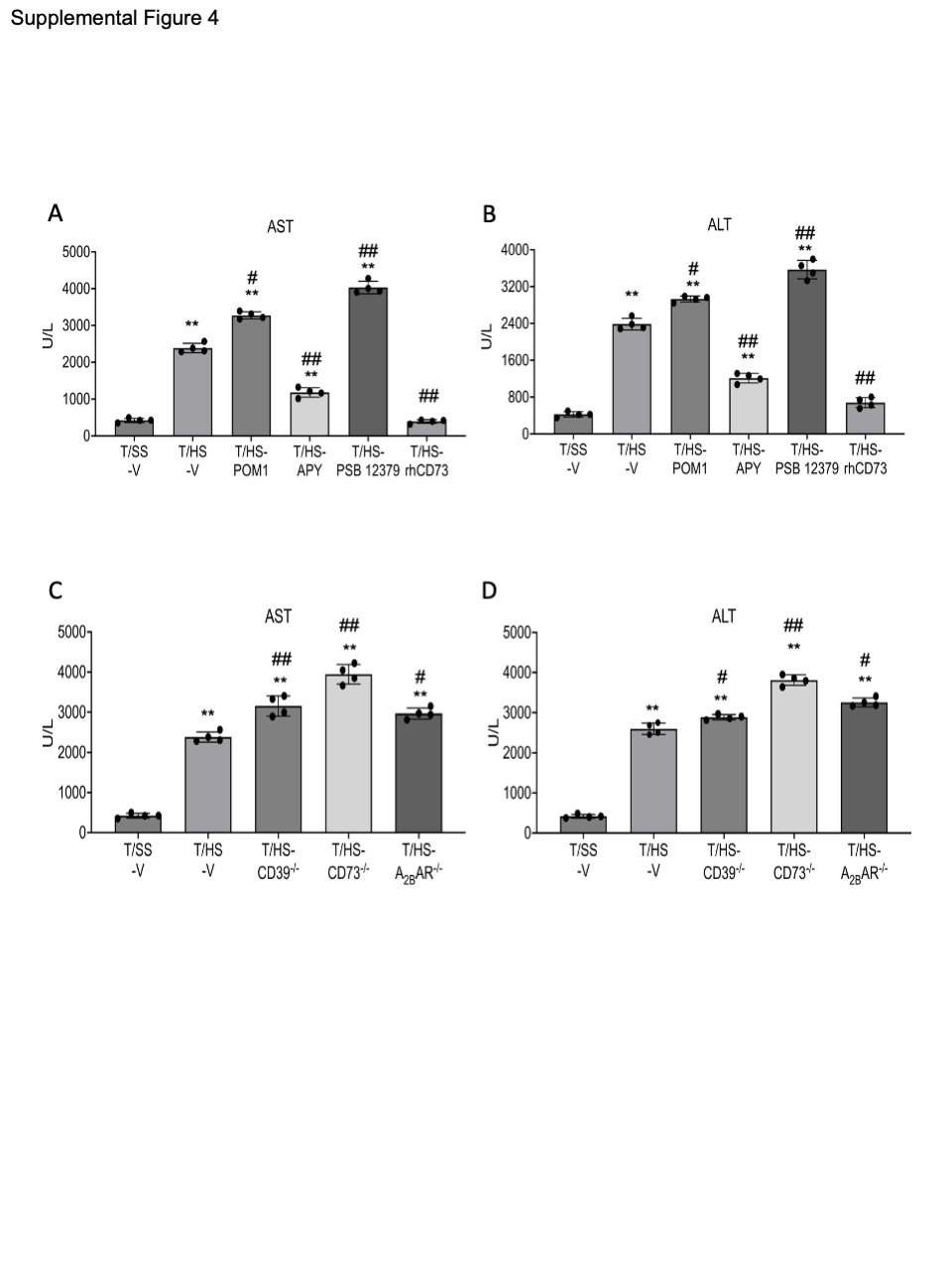

Supplement: Supplementary file 4 — Additional file 4. Fig. S4. CD39, CD73, and A2BR regulation of liver enzymes. Aspartate aminotransferase (AST) (A, C) and alanine aminotransferase (ALT) (B, D) levels were determined from plasma spectrophotometrically. Data are mean ± S.D. (n = 4/group). *p < 0.05 compared with T/HS, **p < 0.05 compared with T/HS, #p < 0.05 compared with corresponding T/HS-V, ##p < 0.01 compared with corresponding T/HS. [file 12931_2023_2486_MOESM4_ESM.tiff]

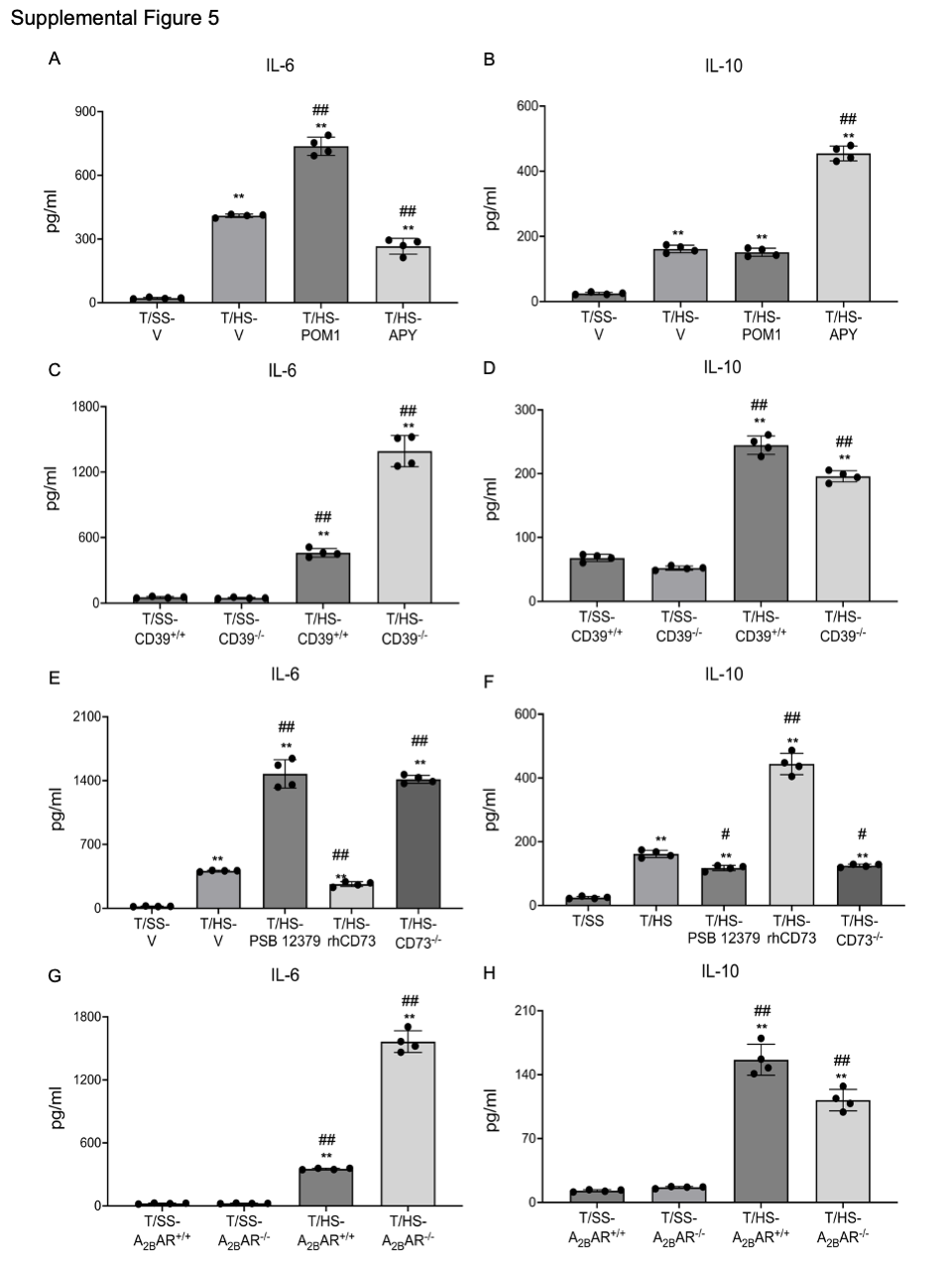

Supplement: Supplementary file 5 — Additional file 5. Fig. S5. Quantification of cytokine levels in plasma. IL-6 (A, C, E, G) and IL-10 (B, D, F, H) in blood 3 h following resuscitation were determined using ELISA. Data are mean ± S.D. (n = 4/group). *p < 0.05 compared with T/HS, **p < 0.05 compared with T/HS, #p < 0.05 compared with corresponding T/HS-V, ##p < 0.01 compared with corresponding T/HS. [file 12931_2023_2486_MOESM5_ESM.tiff]
